# Supplementary material for: WordCommentsAnalyzer: A windows software tool for qualitative research
Source: F1000Res. 2018 Sep 4;7:536. Originally published 2018 May 3. [Version 2] doi: 10.12688/f1000research.14819.2 (PMC6137411; doi:10.12688/f1000research.14819.2)
Supplement: Supplementary file 3 [file f1000research-7-17614-s0002.tgz › b2a79496-4ada-47c4-9682-a99ce867836d.zip!/Eight Iranian Health Journals/Archives of Rehabilitation_11.docx]

© Archives of Rehabilitation (RJ). University of Social Welfare and Rehabilitation Sciences, Koodakyar Alley, Daneshjoo Blv., Evin, Tehran, Iran. This work is licensed under [CC BY-NC 4.0](https://creativecommons.org/licenses/by-nc/4.0/).

Volume & Issue: دوره 16 - شماره 3

Title: بررسی اثربخشی آموزش تمرینهای ادراکی-حرکتی و برنامه حرکتی ریتمیک بر رشد حرکتی در کودکان کمتوان ذهنی آموزش پذیر

http://rehabilitationj.uswr.ac.ir/article-1-1714-fa.html

هدف: هدف از این تحقیق، بررسی اثربخشی آموزش تمرین‌های ادراکی-حرکتی و برنامه حرکتی ریتمیک بر رشد حرکتی کودکان کم‌توان ذهنی آموزش‌پذیر بود.  روش بررسی: در این تحقیق نیمه‌تجربی از نوع پیش آزمون- پس آزمون با گروه کنترل، 30 کودک 10-7 ساله از بین مدارس ابتدایی شهر تبریز به‌صورت تصادفی-خوشه‌ای انتخاب و براساس سن و بهره هوشی همتاسازی شدند و در دو گروه آزمایش (گروه تمرین ادراکی-حرکتی و برنامه حرکتی ریتمیک) و یک گروه کنترل قرارگرفتند. برنامه‌های تمرینی در 9 هفته و هر هفته دو جلسه و هر جلسه 45 دقیقه انجام گرفت. قبل از شروع جلسات و در پایان آخرین جلسه پیش‌آزمون و پس‌آزمون به انجام رسید. به‌منظور سنجش رشد حرکتی از آزمون اولریخ-2 (2000) و برای تجزیه و تحلیل داده‌ها از روش تحلیل کوواریانس چندمتغیره و آزمون تعقیبی بونفرونی به‌کار گرفته شد. تجزیه و تحلیل داده­ها با نرم­افزار SPSS نسخه 18 در سطح معنی داری 0.05>P انجام گرفت.  یافته‌ها: نتایج نشان داد که دو گروه آزمایش (گروه تمرین‌های ادراکی-حرکتی و گروه برنامه حرکتی ریتمیک) در مهارت‌های جابه‌جایی و کنترل شیء نسبت به گروه کنترل عملکرد بهتری داشتند (0/05&leP) و تفاوت بین دو گروه آزمایشی با یکدیگر معنادار نبود (0/05&leP) . در مهارت‌های کنترل شیء، گروه تمرین ادراکی-حرکتی نسبت به دو گروه برنامه حرکتی ریتمیک و کنترل تأثیر بیشتری بر رشد این مهارت‌ها داشت.  نتیجه‌گیری: با توجه به نتایج، برنامه‌های آموزشی مورد استفاده می‌توانند تجربه حرکتی مناسبی برای کودکان باشند. این برنامه‌ها می‌توانند در مدارس با ایجاد فرصت مناسب و ارائه تکالیف متناسب برای تمرین و رشد مهارت‌های حرکتی استفاده شوند.

Title: بررسی ارتباط کارکردهای اجرایی با علائم رفتاری در کودکان مبتلا به اختلال اُتیسم با عملکرد بالا

http://rehabilitationj.uswr.ac.ir/article-1-1481-fa.html

هدف: بررسی رابطه علائم اختلال اتیسم با توانایی‌های شناختی می‌تواند به توصیف بهتر فنوتیپ این اختلال کمک و سبب‌شناسی و درمان اختلال اتیسم را تسهیل نماید. تخریب کارکرد اجرایی یکی از چندین فنوتیپِ شناختی بالقوه در اختلال اتیسم است؛ بنابراین، هدف این مطالعه بررسی رابطه کمبود کارکردهای اجرایی با علائم اختلال اتیسم است. روش بررسی: در این پژوهش مقطعی، 50 کودک مبتلا به اختلال اتیسم عملکرد بالا با استفاده از روش نمونه‌گیری دردسترس از مراکز رشد، به‌آرا و تهران‌پارس انتخاب شدند. سپس، آزمون گارز و پرسشنامه سنجش دامنه اختلال اتیسم توسط درمانگران و آزمون‌های نوروسایکولوژیک عملکرد مداوم، استروپ و توجه انتقالی توسط آزمودنی‌ها تکمیل گردید. به‌منظور تجزیه و تحلیل داده‌ها از ضریب همبستگی پیرسون و تحلیل رگرسیون چندمتغیری استفاده شد. یافته‌ها: نتایج این مطالعه نشان داد بین توجه انتخابی با علائم ارتباطات و تعاملات اجتماعی و بین توجه پایدار با علائم تعاملات اجتماعی و رفتارهای کلیشه‌ای رابطه مثبت و معناداری وجود دارد (0/001>P) و (0/01>P) و (0/05>P) همچنین بین توجه انتقالی با علائم ارتباطات، تعاملات اجتماعی و رفتارهای کلیشه‌ای رابطه مثبت و معنادار وجود دارد (0/001>P) و (0/01>P) و (0/05>P). علاوه‌براین، نتایج تحلیل رگرسیون نشان داد که توجه انتخابی و توجه انتقالی توانایی پیش‌بینی علائم ارتباطات و توجه پایدار توانایی پیش‌بینی علائم تعاملات اجتماعی و رفتارهاری کلیشه‌ای را دارد (0/01>P) و (0/05>P). نتیجه‌گیری : نتایج این مطالعه از نقش کارکردهای اجرایی در علائم اختلال اتیسم حکایت دارد؛ بنابراین، پیشنهاد می‌شود مداخلات درمانی نوین در ترمیم کارکردهای اجرایی در کودکان مبتلا به اختلال اتیسم طراحی شود

Title: بررسی رابطه درد گردن با زاویه سر در بین دانشجویان دختر و پسر با تأکید بر مقاطع تحصیلی مختلف

http://rehabilitationj.uswr.ac.ir/article-1-1585-fa.html

هدف: درد گردن یکی از علل مراجعه جوانان به مراکز کاردرمانی است که ممکن است در اثر وضعیت نامناسب بدنی از جمله قرارگرفتن طولانی‌مدت سر، گردن و شانه‌ها در وضعیت نامناسب حین کارهای روزمره و در طی تحصیل به‌وجود آید. این تحقیق به‌منظور بررسی رابطه درد گردن و زاویه سربه‌جلو در بین دانشجویان دختر و پسر در مقاطع تحصیلی مختلف به انجام رسید. روش بررسی: در این تحقیق همبستگی 1017 دانشجو (511 دختر، 506 پسر) از دانشگاه‌های شهر تهران در مقاطع کارشناسی، کارشناسی ‌ارشد و دکتری شرکت داشتند. انتخاب گروه‌های آموزشی به‌صورت هدفمند ولی انتخاب نمونه‌ها در این گروه‌ها به‌صورت تصادفی بوده است. در دختران میانگین سنی 23.78 ± 3.74 سال، وزنی 63.24±12/1 کیلوگرم و قد 162.42±7.14 سانتی‌متر و پسران میانگین سنی 23/74±3/91 سال، وزنی 57/23±8/87کیلوگرم و قد 168.53±9/74 سانتی‌متر بوده است. دانشجویان توسط پرسشنامه و مقیاس شدت درد (VAS) و گونیامتر مخصوص (HPSCI) برای اندازه‌گیری زاویه سر‌به‌جلو مورد ارزیابی قرار گرفتند. برای بررسی رابطه از آزمون همبستگی پیرسون در نسخه 18 نرم‌افزار SPSS (با سطح معنی‌داری 0/05) استفاده شد. یافته‌ها: رابطه منفی و معنی‌داری بین درد گردن و وضعیت زاویه سربه‌جلو در بین دانشجویان دختر مقطع دکتری به‌دست آمد (P=0/007)؛ درحالی‌که این رابطه در بین دانشجویان دختر و پسر مقطع کارشناسی، کارشناسی‌ ارشد و دانشجویان پسر مقطع دکتری معنی‌دار نشد (P&le0/05). نتیجه‌گیری: با توجه به وجود رابطه بین درد گردن و وضعیت سربه‌جلو دانشجویان دختر در مقطع دکتری، می‌توان در برنامه‌های درمانی بر اصلاح وضعیت‌های نامناسب سر و شانه تأکید کرد. از طرفی به‌دلیل پیشرفت جامعه دانشجویی به‌سمت مقاطع تحصیلی بالاتر، این مسئله برای آگاهی و اطلاع‌رسانی از وضعیت صحیح بدنی دانشجویان اهمیت زیادی دارد.

Title: تأثیر تمرینات تراباند بر حس وضعیت عضلات داخلی و خارجی گرداننده شانه مردان ورزشکار مبتلا به سندرم گیرافتادگی شانه

http://rehabilitationj.uswr.ac.ir/article-1-1440-fa.html

هدف:  این مطالعه به‌منظور بررسی تأثیر تمرینات تراباند بر حس وضعیت عضلات داخلی و خارجی گرداننده شانه مردان ورزشکار مبتلا به  سندرم گیر افتادگی شانه انجام شد. روش بررسی: پژوهش حاضر مداخله‌ای و از نوع تحقیق نیمه‌تجربی به‌صورت طرح پیش‌آزمون-پس‌آزمون با گروه کنترل است که در آن 30 نفر از مردان مبتلا به سندرم گیرافتادگی شانه با دامنه سنی 20 تا 30 سال در دو گروه 15 نفری آزمایش و کنترل به‌صورت غیرتصادفی و هدفدار تقسیم شده بودند، شرکت داشتند. به‌منظور اندازه‌گیری حس وضعیت عضلات داخلی و خارجی گرداننده شانه از دستگاه ایزوکنتیک مدل بایودکس سیستم 3 ساخت کشور آمریکا استفاده شد و برای تجزیه و تحلیل داده‌ها از آزمون تی زوجی و مستقل در نسخه 21 نرم افزار SPSS استفاده شد. یافته‌ها: در گروه آزمایش پس از شش هفته تمرینات تراباند، بهبودی معنی‌داری در حس وضعیت عضلات داخلی و خارجی گرداننده شانه مردان ورزشکار مبتلا به  سندرم گیرافتادگی شانه در سه زاویه 0، 45 و 90 درجه در سطح معنی‌داری 0/05 ایجاد شد (0/05=P). نتیجه‌گیری: نتایج تحقیق حاضر نشان داد که تمرینات تراباند منجر به بهبودی حس وضعیت هر دو گروه عضلانی داخلی و خارجی گرداننده شانه مردان ورزشکار مبتلا به سندرم گیرافتادگی می‌شود؛ از‌این‌رو، از این تمرینات به‌دلیل مزایای آن می‌توان به‌صورت گسترده در تیم‌های ورزشی و همچنین برای توان‌بخشی آسان و سریع‌تر بیماران بهره گرفت.

Title: ساخت و بررسی روایی و پایایی نسخه فارسی «آزمون دایکوتیک کلمات هم‌قافیه آمیخته»

http://rehabilitationj.uswr.ac.ir/article-1-1691-fa.html

هدف: پژوهش حاضر با هدف ساخت و بررسی روایی و پایایی نسخه فارسی آزمون دایکوتیک کلمات هم‌قافیه آمیخته در جمعیت هنجار 25-18 ساله فارسی‌زبان انجام گرفته است. روش بررسی: نسخه فارسی آزمون حاضر، از 15 جفت کلمه تک‌هجایی هم‌قافیه تشکیل و در قالب 4 لیست 30 تایی تنظیم شده است. کلمات هر جفت به‌طور هم‌زمان یکی به گوش چپ و دیگری به گوش راست ارائه و منجر به درک یک مفهوم آمیخته منفرد می‌شوند. پس از انتخاب مواد آزمون از فرهنگ فارسی معین براساس معیارهای موردنظر و جفت‌کردن کلمات هم‌قافیه، روایی محتوایی با نظرسنجی از 10 کارشناس خبره و از طریق روش لاوشه به‌دست آمد و جفت‌کلمات با روایی بالا انتخاب و فهرست آزمون تنظیم گردید. سپس، کلمات هر فهرست به صورت دایکوتیک روی لوح فشرده ضبط گردید. درادامه، مطالعه حاضر روی 124 فرد هنجار راست‌دست (68 زن و 56 مرد) در محدوده سنی 25-18 سال اجرا و نتایج در فرم‌های امتیازدهی طراحی‌شده ثبت گردید. به‌منظور بررسی پایایی آزمون پس از دو هفته از اجرای اولیه، 15 نفر از افراد دوباره مورد ارزیابی قرارگرفتند. یافته‌ها: بین میانگین امتیازات گوش راست و چپ تفاوت معنادار مشاهده شد (0.001>P). شاخص روایی محتوا (CVR) برای هر آیتم آزمون بین 0.8 تا 1، ضریب همبستگی پیرسون برای آزمون-بازآزمون 0.38  (0.001>P) و آلفای کرونباخ و ضریب همبستگی درون‌رده‌ای (ICC) برای پایایی بین لیست‌های آزمون به‌ترتیب0.81 و  0.84 به‌دست آمد و بین لیست‌ها همبستگی معنی‌دار مشاهده گردید. نتیجه‌گیری: براساس نتایج به‌دست‌آمده، نسخه فارسی آزمون حاضر از روایی محتوایی و پایایی خوبی برخوردار است و می‌تواند در تشخیص عملکرد جسم پینه‌ای، سوبرتری نیمکره‌های مغزی و ارزیابی پردازش شنیداری مفید باشد.

Title: تأثیر خستگی عضلانی بر بیومکانیک طبیعی کمربند شانهای: مروری نظاممند بر مطالعات گذشته

http://rehabilitationj.uswr.ac.ir/article-1-1453-fa.html

هدف: ثبات دینامیک در شانه، جزیی حیاتی است و هرگونه اختلال در عملکرد عضلاتی که به حفظ این ثبات کمک می‌کند منجر به پاتولوژی می‌شود. یکی از عوامل برهم‌زننده عملکرد عضلات، خستگی است. ازآنجایی‌که برطبق اطلاعات گروه تحقیق تا‌به‌حال پژوهش جامعی این مطالعات را مورد بررسی قرار نداده است؛ بنابراین، هدف از انجام این مطالعه مروری نظام‌مند، بررسی تأثیر خستگی عضلانی بر بیومکانیک طبیعی کمربند شانه‌ای است. روش بررسی: این مطالعه براساس جستجوی الکترونیکی در بانک‌های اطلاعاتی پاب‌مد (PubMed)، اِسکوپوس (Scopus)، اِلزویر (Elsevier)، اُوید (Ovid)، سیناهِیل (CINAHL)، سایِنس دایرِکت (Science Direct)، پِروکوییست (ProQuest)، تامپسون (Thompson)، اِمبِیس (EMBASE) و مِدلاین (Medline) در فاصله زمانی بین سال‌های 2012-1995 انجام گرفته است. کلیدواژه‌های استفاده‌شده عبارت از شانه، خستگی عضلانی، حس عمقی و کینماتیک بود. یافته‌ها: حاصل این جستجوی الکترونیکی در بانک‌های اطلاعاتی مذکور، 350 مقاله بود که 22 مطالعه معیارهای لازم را برای ورود به مطالعه داشتند. تفاوت‌های بین مطالعات از نظر تعداد نمونه‌ها، پروتکل خستگی و روش‌های اندازه‌گیری مورد بررسی قرار گرفتند. نتیجه‌گیری: بررسی مقالات نشان داد که خستگی فعالیت عضلات کمربند شانه‌ای و درنتیجه کینماتیک ناحیه را تغییر می‌دهد و همچنین منجر به کاهش حس عمقی در طی وضعیت‌ها و حرکات مختلف در هر دو مفاصل اسکاپولوتراسیک و گلنوهومرال می‌شود. به‌طورکلی، نتایج نشان داد که شواهد کافی مبنی بر تأثیر خستگی بر کینماتیک کمربند شانه‌ای وجود دارد و بیومکانیک طبیعی شانه را تغییر می‌دهد.

Title: ارتباط مهارتهای ادراک بینایی غیروابسته به حرکت با مهارت خواندن در دانش‌آموزان فلج مغزی اسپاستیک

http://rehabilitationj.uswr.ac.ir/article-1-1560-fa.html

هدف: هدف از تحقیق حاضر، بررسی و تعیین ارتباط زیرمجموعه‌های ادراک بینایی با مؤلفه‌های دقت و سرعت مهارت خواندن دانش‌آموزان فلج مغزی پایه دوم دبستان است. روش بررسی : در این مطالعه مقطعی و تحلیلی، تعداد 24 نفر از دانش‌آموز فلج مغزی پایه دوم دبستان (10 دختر و 14 پسر) به‌صورت دردسترس از کلینیک‌ها و مراکز توان‌بخشی شهر تهران براساس مناطق جغرافیایی انتخاب شدند. مهارت‌های ادراک بینایی، با آزمون مهارت‌های ادراک بینایی غیروابسته به حرکت (TVPS-R) و مهارت خواندن نیز به‌وسیله آزمون تشخیصی خواندن مورد سنجش قرار گرفت. نتایج به‌دست‌آمده پس از بررسی نوع توزیع متغیرها براساس آزمون کولوموگروف-اسمیرونوف و با استفاده از آزمون‌های غیرپارامتریک اسپیرمن با نسخه 18 نرم‌افزار آماری SPSS مورد تجزیه و تحلیل قرار گرفتند. یافته‌ها: یافته‌های این مطالعه در آزمون همبستگی اسپیرمن نشان داد که بین نمرات استاندارد هفت حوزه ادراک بینایی با دو مؤلفه مهارت خواندن در دانش‌آموزان فلج مغزی رابطه معناداری وجود دارد. مؤلفه‌های تمییز بینایی، حافظه بینایی، روابط فضایی-بینایی، ثبات شکل بینایی، حافظه توالی بینایی، شناخت شکل از زمینه و اکمال بینایی با دقت و سرعت در ارتباط است (05/P<0). نتیجه‌گیری:  ارتباط هر یک از مهارت‌های ادراک بینایی با دقت و سرعت مهارت خواندن دانش‌آموزان فلج مغزی کلاس دوم دبستان مشهود و معنادار است و می‌توان چنین نتیجه گرفت که به‌منظور بهبود مهارت خواندن دانش‌آموزان فلج مغزی در زمینه‌های دقت و سرعت، می‌توان از این تمرینات ادراک بینایی استفاده نمود.

Title: عوامل مؤثر بر مشارکت دانشجویان در فرایند یاددهی-یادگیری از دیدگاه اساتید

http://rehabilitationj.uswr.ac.ir/article-1-1497-fa.html

هدف: هدف این مطالعه شناسایی عوامل مؤثر بر مشارکت دانشجویان در فرایند یاددهی-یادگیری از دیدگاه اساتید دانشگاه علوم بهزیستی و توانبخشی تهران است. روش بررسی: این مطالعه، مطالعه‌ای توصیفی است که از روش نمونه‌گیری تمام‌شماری و با استفاده از پرسشنامه محقق‌ساخته بر روی 116 نفر از اساتید انجام پذیرفت. سپس، داده‌های حاصل از پژوهش از طریق آزمون تحلیل واریانس تک‌متغیری با اندازه‌گیری‌های مکرر آزمون بونفرونی مورد تجزیه و تحلیل قرار گرفته است. یافته‌ها: یافته‌های این مطالعه بیانگر آن است که بین میانگین نمرات اساتید در هر یک از حیطه‌های «مشارکت دانشجویان» (با میانگین 23/2) و انحراف استاندارد (با میانگین 2/8)، «کیفیت ارتباطات میان‌فردی دانشجویان» (با میانگین 25/1) و انحراف استاندارد (با میانگین 4/1)، «سبک تفکر اساتید» (با میانگین 24/2) و انحراف استاندارد (با میانگین 4/3)، «کیفیت زندگی کاری اساتید» (با میانگین 23/3) و انحراف استاندارد (با میانگین 4/1) و «نگرش اعضای هیئت علمی نسبت به عوامل حرفه‌ای» (با میانگین 26/1) و انحراف استاندارد (با میانگین 3/1) در زمینه مشارکت دانشجویان در فرایند یاددهی-یادگیری تفاوت معنادار وجود دارد (0/05>P). نتیجه‌گیری: با توجه به نتایج مطالعه می‌توان چنین عنوان کرد که حیطه‌های «نگرش اساتید نسبت به عوامل حرفه‌ای» و «کیفیت ارتباطات میان‌فردی دانشجویان» بیشترین تأثیر را بر مشارکت دانشجویان در فرایند یاددهی–یادگیری دارند.
